# hucMSC-derived exosomes attenuate colitis by regulating macrophage pyroptosis via the miR-378a-5p/NLRP3 axis

**DOI:** 10.1186/s13287-021-02492-6

**Published:** 2021-07-22

**Authors:** Xiu Cai, Zhi-yu Zhang, Jin-tao Yuan, Dickson Kofi Wiredu Ocansey, Qiang Tu, Xu Zhang, Hui Qian, Wen-rong Xu, Wei Qiu, Fei Mao

**Affiliations:** 1 Key Laboratory of Medical Science and Laboratory Medicine of Jiangsu Province, School of Medicine, Jiangsu University, Zhenjiang, Jiangsu People’s Republic of China; 2 The People’s Hospital of Danyang, Affiliated Danyang Hospital of Nantong University, Zhenjiang, Jiangsu 212300 People’s Republic of China; 3 Directorate of University Health Services, University of Cape Coast, Cape Coast, Ghana; 4 Nanjing Jiangning Hospital, Nanjing, Jiangsu 211100 People’s Republic of China

**Keywords:** hucMSC-derived exosomes, miR-378a-5p, IBD, NLRP3, Macrophage, Pyroptosis

## Abstract

**Background:**

Human umbilical cord mesenchymal stem cell (hucMSC)-derived exosomes are recognized as novel cell-free therapeutic agents for inflammatory bowel disease (IBD), a condition caused by dysregulated intestinal mucosal immunity. In this event, macrophage pyroptosis, a process of cell death following the activation of NLRP3 (NOD-like receptor family, pyrin domain-containing 3) inflammasomes, is believed to partially account for inflammatory reactions. However, the role of macrophage pyroptosis in the process of hucMSC-derived exosomes alleviating colitis remains unknown. This study aimed at exploring the therapeutic effect and mechanism of hucMSC-derived exosomes on colitis repair.

**Methods:**

In vivo, we used BALB/c mice to establish a dextran sulfate sodium (DSS)-induced colitis model and administrated hucMSC-derived exosomes intravenously to estimate its curative effect. Human myeloid leukemia mononuclear (THP-1) cells and mouse peritoneal macrophages (MPMs) were stimulated with lipopolysaccharides (LPS) and Nigericin to activate NLRP3 inflammasomes, which simulated an inflammation environment in vitro. A microRNA mimic was used to verify the role of miR-378a-5p/NLRP3 axis in the colitis repair.

**Results:**

hucMSC-derived exosomes inhibited the activation of NLRP3 inflammasomes in the mouse colon. The secretion of interleukin (IL)-18, IL-1β, and Caspase-1 cleavage was suppressed, resulting in reduced cell pyroptosis. The same outcome was observed in the in vitro cell experiments, where the co-culture of THP-1 cells and MPMs with hucMSC-derived exosomes caused decreased expression of NLRP3 inflammasomes and increased cell survival. Furthermore, miR-378a-5p was highly expressed in hucMSC-derived exosomes and played a vital function in colitis repair.

**Conclusion:**

hucMSC-derived exosomes carrying miR-378a-5p inhibited NLRP3 inflammasomes and abrogated cell pyroptosis to protect against DSS-induced colitis.

## Background

IBD, encompassing Crohn’s disease (CD) and ulcerative colitis (UC), is a complex chronic inflammatory disorder associated with multiple pathogenic factors including environmental changes, genetic susceptibility, qualitative and quantitative abnormal gut microbiota, and dysregulated immune response [1, 2]. IBD has evolved into a global disease with rising global prevalence, including newly industrialized countries in Asia [3]. Available evidence in the last decades suggests that the dysfunction of innate and adaptive immune pathways causes aberrant inflammation in patients with IBD [4, 5]. In gut homeostasis maintenance, macrophages are seen as one of the main players that sense the microbe-associated molecular patterns (MAMPs) via innate immune receptors such as Toll-like receptors (TLRs) and nucleotide-binding domain and leucine-rich-repeat receptors (NLRs) [6]. Pro-inflammatory cytokines like IL-1β, IL-6, and tumor necrosis factor (TNF)-α produced by intestinal macrophages result in immune cell activation and trigger mucosal inflammation, which has broad implications in the early stage of IBD. At present, macrophages are increasingly recognized as the gatekeepers of intestinal immune homeostasis and it has been considered as a novel target to develop new therapies [7].

Mesenchymal stem cells (MSCs) play an immunomodulatory and homeostatic role in inflammation and offer a strategy for promoting tissue repair of inflammatory-induced injury [8]. Emerging evidence shows that MSCs perpetuate immunosuppressive signaling via secreting paracrine mediators rather than cell-to-cell contact [9]. Thus, MSC-derived exosomes mediating paracrine effects attribute to their therapeutic effect. Exosomes transfer bioactive cargo such as lipids, functional microRNAs (miRNAs), messenger RNAs (mRNAs), and proteins, mediating specific intracellular signaling pathways [10, 11]. MSC-derived exosomes have suppressive effects on innate immune cells like dendritic cells (DCs), monocytes, and macrophages and alleviate colonic inflammation [12]. A number of published researches indicate that MSC-derived exosomes can alleviate colonic inflammation [13–16]. Liu et al. reported that MSC-derived exosomes downregulated colonic inflammatory responses, maintained intestinal barrier integrity, and polarized macrophages to M2b phenotype to reduce murine experimental colitis [14], offering a feasible and promising cell-free therapeutic strategy for IBD.

Inflammasomes have been demonstrated to be associated with immune and inflammation-related disorders in many systems, covering myocardial infarction, atherosclerosis, IBD, diabetes, and immune diseases [17–19]. NLRP3 inflammasome, the most well-studied, is a cytosolic protein complex comprised of pattern recognition receptor NLRP3, adaptor protein apoptosis-associated speck-like protein containing a caspase recruitment domain (ASC) and pro-Caspase-1 [20]. Under the stimulation of pathogen-associated molecular patterns (PAMPs) and danger-associated molecular patterns (DAMPs), the pattern recognition receptors recognize endogenous and exogenous signals. This triggers the assembly of the inflammasomes and the cleavage of Caspase-1, which leads to the maturation and secretion of pro-IL-1β and pro-IL-18 [21]. Besides, the NLRP3 inflammasomes initiate pyroptosis by activating the cleavage of gasdermin D (GSDMD) which forms pores in the plasma membrane [22]. The overactivation of NLRP3 inflammasomes may aggravate the onset and development of inflammatory diseases [23]; hence, its inhibition serves as a therapeutic target in attenuating IBD [24]. Therefore, we hypothesized that hucMSC-derived exosomes may relieve colonic inflammation by inhibiting NLRP3 inflammasomes.

In this study, we aimed at mitigating colitis in mice by the use of hucMSC-derived exosomes. After determining the therapeutic role of hucMSC-derived exosomes in IBD, the miRNA sequence between hucMSC-derived exosomes and HFL-1-derived exosomes was analyzed to predict potential miRNA targeting NLRP3 to unravel possible molecular mechanisms of the putative anti-inflammatory effect of hucMSC-derived exosomes.

## Methods

The study was approved by the Ethical Committee of Jiangsu University (2012258).

### Cell culture

hucMSCs were isolated from a fresh umbilical cord (in addition to the ethical approval obtained for the study, parturients agreed to the use of their umbilical cords for the study) as previously described [25] and cultured in α**-**MEM medium (Invitrogen, Grand Island, NY, USA). Human myeloid leukemia mononuclear cells (THP-1 cells) were purchased from Beiner Biotechnology Company (Beijing, China) and cultured in RPMI 1640 medium (Invitrogen) containing 10% fetal calf serum (FBS; BioInd, Israel) at 37 °C in humid air with 5% CO^2^.

### Exosomal extraction

Exosomes were extracted and purified as previously described [26]. The protein content of isolated exosomes was quantified using a BCA protein assay kit (CWBIO, Beijing, China). The concentration of hucMSC-derived exosomes for in vitro use was 200 μg ml^−1^ and a total of 1 mg of exosomes was applied to treat each mouse. The concentration and size distribution of hucMSC-derived exosomes were analyzed by NanoSight Nano Analyzer (Malvern Panalytical, Malvern, UK). The morphology of hucMSC-derived exosomes was observed by transmission electron microscopy (Philips, Amsterdam, The Netherlands). Exosomal protein markers CD9, CD63, CD81, HSP70, Alix, and Calnexin were determined by western blot.

### Animal model and treatment

Male BALB/c mice (6–8 weeks old, 20 ± 2 g) were purchased from the Animal Research Center of Jiangsu University (Zhenjiang, China). Mice were randomly allocated into three groups (*n* = 6/group): the negative control group (NEG group), DSS-induced colitis group (IBD group), and hucMSC-derived exosome-treated colitis group (hucMSC-Ex group). Mice in the NEG group were fed autoclaved water throughout the study, while mice in the IBD group and the hucMSC-Ex group were treated with 3% DSS (MP Biomedicals, CA, USA) dissolved in autoclaved water. One milligram of hucMSC-derived exosomes was administrated by caudal vein injection to mice in the hucMSC-Ex group on the 3rd, 6th, and 9th day, while mice in other groups were injected with PBS through the tail vein. Mouse body weight was recorded at the same time every day, fecal chrematistics monitored, and disease activity index (DAI) analyzed as previously described [27]. All the mice were sacrificed on the 11th day and colons were collected for observation.

### Animal live imaging analysis

hucMSC-derived exosome (1 mg) was stained with 1 μg fluorochrome DIR (Thermo Fisher Scientific, Waltham, MA, USA) by incubating on a shaker at 37 °C for 30 min, followed by centrifuging at 12000g. The hucMSC-derived exosomes-DIR complex diluted in PBS was intravenously injected into the mice in the hucMSC-Ex group. Twelve hours later, the fluorescence distribution in the mice was tracked via a live imaging system (IVIS@ Lumina LT Series III; PerkinElmer, Waltham, MA, USA) (*λ*/nm = 720) [28]. The mice’s colorectal tissues were observed for the fluorescence location after mice were sacrificed.

### Isolation of mouse peritoneal macrophages (MPMs)

Peritoneal macrophages were isolated as described previously [29]. Mice were sacrificed by cervical dislocation and immersed in 75% ethanol for 5 min for sterilization. A sterile scalpel was used to make an incision on the mice’s abdomen to expose the peritoneum. Peritoneal lavage was performed with 5 ml sterile PBS containing 3% FBS and cells were centrifuged at 310g for 5 min at 4 °C. The supernatant was discarded and cells were resuspended in RPMI 1640 with 10% FBS, followed by seeding in a 6-well plate allowing macrophages to attach for 3 h.

### Macrophage differentiation and stimulation

THP-1 cells were induced to macrophage-like phenotype by the addition of 50 ng ml^−1^ PMA (Sigma Aldrich, St. Louis, MO, USA) for 16 h before cell experiments. The differentiated THP-1 cells and MPMs were set up into three groups: the negative control (Ctrl), LPS + NIG (LPS + NIG), and LPS + NIG + hucMSC-Ex (hucMSC-Ex). One microgram per milliliter LPS (Sigma Aldrich) was added to the LPS + NIG group and hucMSC-Ex group, followed by the addition of 1 μM Nigericin (Invitrogen) after 4 h, while 200 μg ml^−1^ hucMSC-derived exosome was added to the hucMSC-Ex group at the same time of LPS addition. After 12 h of co-cultivation, cells and supernatants were collected for subsequent analysis.

### Hematoxylin and eosin (H&E) staining

A portion of mice’s colorectal tissue was fixed with 4% paraformaldehyde (PH 7.4) and gradually dehydrated, embedded in paraffin, cut into 4-μm sections, and stained with H&E, followed by mounting and scanning with pathological section scanner.

### Immunohistochemistry (IHC)

Paraffin-embedded colorectal tissues of mice were dewaxed and exposed to 3% hydrogen peroxide for 30 min at room temperature to inhibit endogenous peroxidase activity. Tissues were steamed for 30 min in citrate buffer to repair antigens and incubated with 5% bovine serum albumin (BSA) solution to block non-specific antigens. Primary antibodies such as NLRP3 (1:100; Novus, CO, USA), IL-1β (1:100; Cell Signaling Technology, MA, USA), Caspase-1 p45 (1:100; Proteintech Group, Chicago, IL, USA), and Caspase-1 p20 (1:50; Santa Cruz Biotechnology, CA, USA) were added for overnight incubation at 4 °C, followed by the secondary antibody (Wuhan Boster Biological Technology, Wuhan, China) at 37 °C for 30 min. Then, StreptAvidin Biotin Complex (SABC) was added and incubated at 37 °C for 30 min. Finally, diaminobenzidine substrate (DAB) was applied to sections and counterstained with hematoxylin for microscopic examination after resin sealing.

### Western blot analysis

RIPA lysate (Thermo Fisher Scientific, MA, USA) was applied to colon tissues and cells and protein concentration was measured by the BCA method. Protein samples (200 μg) were separated on a 12% sodium dodecyl sulfate-polyacrylamide gel electrophoresis (SDS-PAGE). The protein was transferred to a PVDF membrane (Millipore, Billerica, MA, USA) and blocked with 5% skim milk to reduce non-specific antigens exposure. The PVDF membranes were incubated with the primary antibodies: anti-CD9 (1:500; Proteintech Group), anti-CD63 (1:500; Abcam, Cambridge, UK), anti-CD81 (1:500; Proteintech Group), anti-HSP70 (1:500; Abcam), anti-Alix (1:500; Cell Signaling Technology), anti-Calnexin (1:500; Abcam), anti-NLRP3 (1:1000; Novus), anti-ASC (1:1000; Novus), anti-Caspase-1 p45 (1:1000; Proteintech Group), anti-Caspase-1 p20 (1:200; Santa Cruz Biotechnology), anti-IL-1β (1:500; Cell Signaling Technology), anti-IL-18 (1:200; Wanleibio, Shenyang, China), anti-GSDMD (1:500; Cell Signaling Technology), and anti-β-actin (1:10000; Abclonal, Boston, MA, USA) at 4 °C overnight, followed by the HRP-conjugated secondary antibodies for 30 min at 37 °C. A chemical gel imaging system (GE Healthcare Life sciences China, Beijing, China) was used to visualize protein bands and generate images.

### Real-time fluorescence quantification PCR (RT-PCR)

RNAs were obtained from mouse colon and cells using Trizol Reagent (Gibco, MA, USA) via chloroform extraction. A reverse transcription kit (Vazyme, Nanjing, China) was used to get the cDNA. The target gene expression was determined by RT-PCR in a Step One Plus Real-time PCR System (ABI, Carlsbad, CA, USA). β-actin was used as an internal control. The sequences of primers used are shown in Table 1.

**Table 1:** Primer sequences for RT-PCR

Species	Gene	Primer sequence	Temperature
Mouse	β-actin	For:TGGAATCCTGTGGCATCCATGAAAC Rev:TAAAACGCAGCTCAGTAACAGTCCG	60 °C
	NLRP3	For:GAGCTGGACCTCAGTGACAATGC Rev:ACCAATGCGAGATCCTGACAACAC	60 °C
	ASC	For:ACAATGACTGTGCTTAGAGACA Rev:CACAGCTCCAGACTCTTCTTTA	58 °C
	Caspase-1	For:AGAGGATTTCTTAACGGATGCA Rev:TCACAAGACCAGGCATATTCTT	58 °C
	IL-1β	For:TCGCAGCAGCACATCAACAAGAG Rev:AGGTCCACGGGAAAGACACAGG	60 °C
	IL-18	For:AGACCTGGAATCAGACAACTTT Rev:TCAGTCATATCCTCGAACACAG	60 °C
	IL-6	For:AAGTCCGGAGAGGAGACTTC Rev:TGGATGGTCTTGGTCCTTAG	58 °C
	IL-10	For:TTCTTTCAAACAAAGGACCAGC Rev:GCAACCCAAGTAACCCTTAAAG	60 °C
	TNF-α	For:AACTCCAGGCGGTGCCTATG Rev:TCCAGCTGCTCCTCCACTTG	63 °C
Human	β-actin	For:CTCAGGAGGAGCAATGATCT Rev:GACCTGTACGCCAACACAGT	60 °C
	NLRP3	For:GCACTTGCTGGACCATCCTC Rev:GTCCAGTGCACACGATCCAG	60 °C
	ASC	For:CTCAAGAAGTTCAAGCTGAAGC Rev:TAGGTCTCCAGGTAGAAGCTG	58 °C
	Caspase-1	For:GAAGAAACACTCTGAGCAAGTC Rev:GATGATGATCACCTTCGGTTTG	58 °C
	IL-1β	For:GCCAGTGAAATGATGGCTTATT Rev:AGGAGCACTTCATCTGTTTAGG	60 °C
	IL-18	For:GCTGAAGATGATGAAAACCTGG Rev:CAAATAGAGGCCGATTTCCTTG	60 °C

### Immunofluorescence (IF)

THP-1 cells were fixed with 4% paraformaldehyde (PH 7.4) at room temperature for at least 20 min and ruptured with 0.1% Triton-X-100 for 30 min. Non-specific antigens were blocked with a 5% BSA solution. Cells were incubated with anti-ASC (1:50; Novus) at 4 °C overnight and then incubated with diluted fluorescent secondary antibody at 4 °C for 1 h. The nuclei were counterstained with hoechest33342 (1:300; Sigma Aldrich) for 15 min at room temperature, followed by an anti-quenching agent. Images were taken with a confocal laser microscope (Nikon, Tokyo, Japan). Exposure to light was avoided throughout the whole process.

### Propidium iodide staining

Cells treated with corresponding reagents were washed with PBS and trypsinized to obtain a single-cell suspension. The concentration of 10^5^ cells/ml was suspended in a 100-μl binding buffer along with 2 μl propidium iodide (Sigma Aldrich) for 10 min at 4 °C, followed by an acquisition using the FACS Calibur (Beckman Coulter, CA, USA). The results were analyzed using CytExpert software.

### CCK8

Cells were divided into a 96-well plate and treated with LPS and Nigericin as described above. CCK8 solution (Vazyme) was added to culture wells and incubated for 30 min in the dark. Absorbance was measured at 450 nm with a microplate reader (Thermo Fisher Scientific).

### miR-378-5p mimics and inhibitor transfection

The transfection solution was constituted according to the manufacturer’s instructions. THP-1 cells were allocated into a six-well plate with the following groups: negative control (Ctrl), LPS + NIG (LPS + NIG), LPS + NIG + mimics (LPS + NIG + mimics), LPS + NIG + mimics NC (LPS + NIG + mimics NC), LPS + NIG + inhibitors (LPS + NIG + inhibitors), and LPS + NIG + inhibitors NC (LPS + NIG + inhibitors NC). After cell differentiation, the original culture medium was discarded, followed by the addition of RPMI 1640 nutrient solution without FBS. The cells were cultured in a 5% CO^2^ incubator in the dark for 6 h; then, the culture medium without FBS was replaced with a normal RPMI 1640 nutrient solution containing 10% FBS. To each well was added 1 μg ml^−1^ of LPS apart from the Ctrl group well, followed by the addition of 1 μM Nigericin after 4 h. After co-culture for 12 h, the cells were collected for subsequent analysis.

### Luciferase reporter assay of miRNA target

The 3′UTR region of NLRP3 mRNA containing the miR-378a-5p binding site, wild or mutant (TCAGGAA mutated to ATTTGCC), was cloned into a dual-luciferase miRNA target expression vector (GP-miRGLO) (GenePharma, Shanghai, China). With the empty vector as the control group, the wild and mutant vectors were co-transfected into HEK293T cells with miR-378a-5p mimics and mimic NC. The Firefly and Renilla luciferase activities were measured by the Dual-luciferase Reporter Assay (Promega, Madison, WI, USA).

### Statistical analysis

All data were shown as mean ± standard deviation (SD). Statistical analysis was performed using GraphPad Prism software (GraphPad Software, San Diego, CA, USA). Comparisons between multiple groups were assessed by one-way ANOVA with the Bonferroni post hoc test. *P* < 0.05 was considered significant.

## Results

### Characterization of hucMSC-derived exosomes

**Fig. 1:**
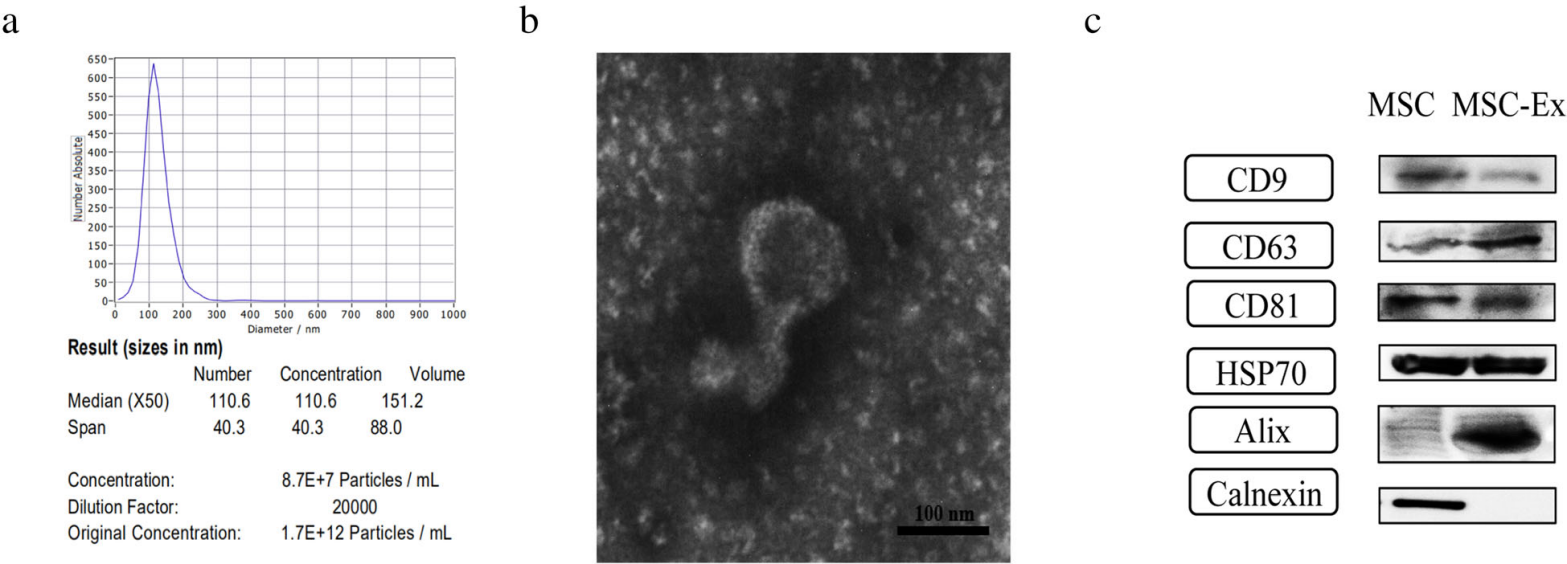
Identification of hucMSC-derived exosomes. **a** NanoSight Nanoparticle Tracking Analyzer detection of hucMSC-derived exosome diameters. **b** Transmission electron microscope observation of hucMSC-derived exosome morphology. **c** Western blot analysis of hucMSC-derived exosome protein markers

The identity and purity of nanoparticles were determined by nanoparticle tracking analysis (NTA), transmission electron microscopy (TEM), and western blot. The results of NTA and TEM showed that hucMSC-derived exosomes exhibited a typical vesicle structure with an average diameter of approximately 110 nm (Fig. 1A, B). Exosomal surface marker proteins CD9, CD63, CD81, HSP70, and Alix were all expressed but negative for Calnexin in the western blot (Fig. 1C).

### hucMSC-derived exosomes alleviate DSS-induced murine colitis

**Fig. 2:**
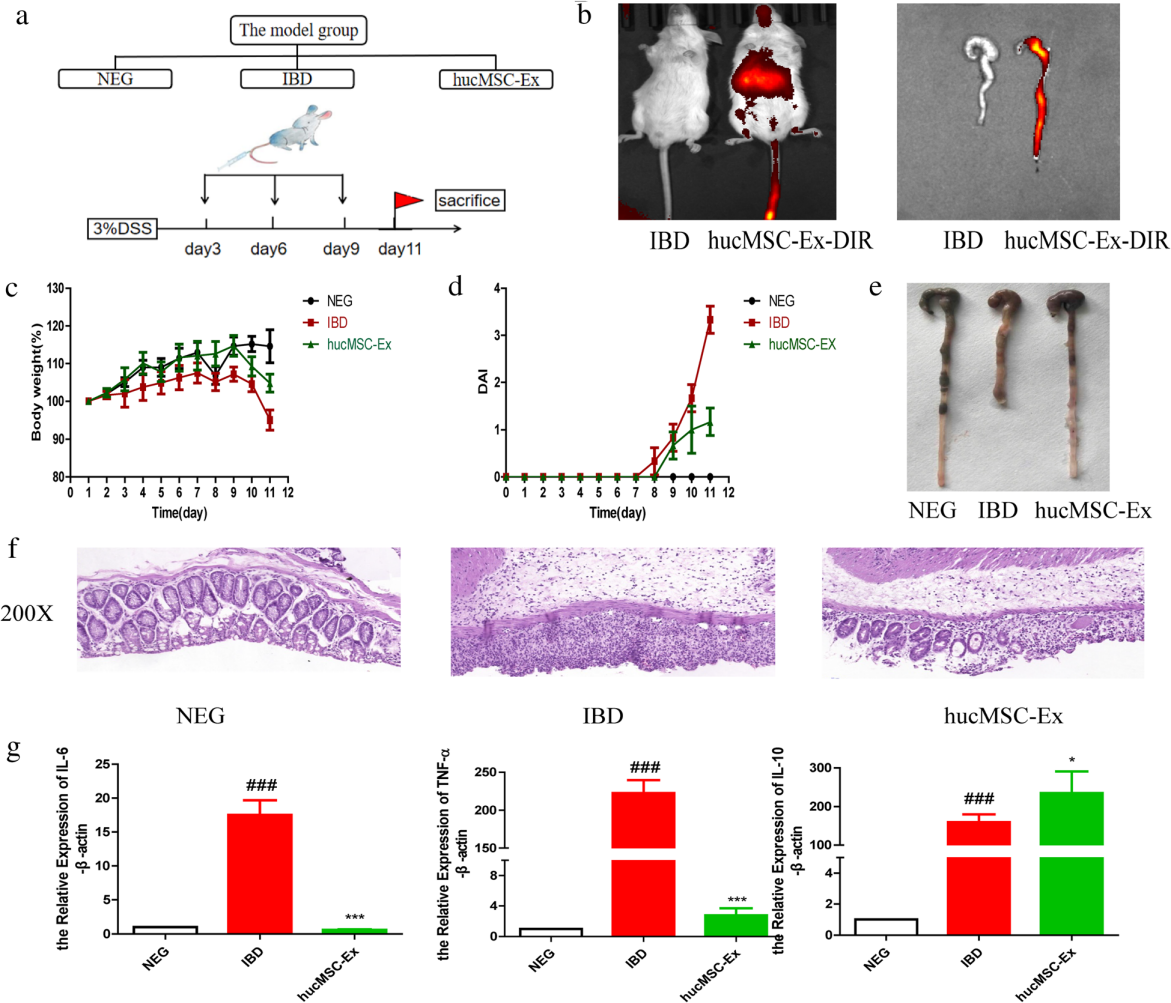
hucMSC-derived exosomes alleviate DSS-induced mice colitis. **a** Colitis model established. The model contained the control (Neg), IBD, and hucMSC-Ex groups. 3% DSS was given at day 1 to mice in the IBD group and hucMSC-Ex group. Mice in the hucMSC-Ex group were given hucMSC-derived exosomes *iv.* at days 3, 6, and 9. **b** Fluorescence distribution of DIR-labeled hucMSC-derived exosomes in mouse colon after injection. **c** The mouse body weight loss. **d** DAI index of mice. **e** Mouse colon appearance. **f** H&E staining of mouse colon (200×, scale bar = 50 μm). **g** QRT-PCR analysis of the expression of inflammatory cytokines (IL-6, TNF-α, IL-10) in mouse colon tissues. #*P* < 0.05, ##*P* < 0.01, ###*P* < 0.001 vs Ctrl by ANOVA; *P < 0.05, **P < 0.01, ***P < 0.001 vs LPS + NIG by ANOVA

To investigate the therapeutic value of hucMSC-derived exosomes in mice colitis, a model group was designed as shown in Fig. 2A. An assessment of successful gut homing of hucMSC-derived exosomes was carried out by tracking fluorescent dye (DIR)-labeled hucMSC-derived exosomes in mice after caudal vein injection. hucMSC-derived exosomes were found to successfully target damaged colorectal tissues at 12 h post-injection (Fig. 2B). The administration of hucMSC-derived exosomes relieved mouse weight loss (Fig. 2C) and reduced DAI (Fig. 2D), with mice in the IBD group exhibiting blood stool earlier than mice in the hucMSC-Ex group. On the 11th day after colitis establishment, all mice were sacrificed. The colon length of the IBD group was the shortest, while the treatment of hucMSC-derived exosomes recovered colon length close to that of the negative group (Fig. 2E). H&E staining confirmed large areas of colorectal tissue disorder without the normal intestinal gland in the IBD group, while hucMSC-derived exosomes significantly ameliorated DSS-induced intestinal injury and restored the structural integrity of colonic mucosal tissue (Fig. 2F). The relative expression of pro-inflammatory cytokines (IL-6, TNF-α) and anti-inflammatory cytokine (IL-10) via RT-PCR indicated that pro-inflammatory cytokines were increased in colorectal tissues of the IBD group, but significantly decreased in the hucMSC-Ex group, while the IL-10 level was higher in the hucMSC-Ex group than in the IBD group (Fig. 2G). Thus, hucMSC-derived exosomes relieve mouse colonic inflammation and promote the repair of the damaged colon in DSS-induced murine colitis.

### hucMSC-derived exosomes decrease NLRP3 inflammasomes to ease inflammation

**Fig. 3:**
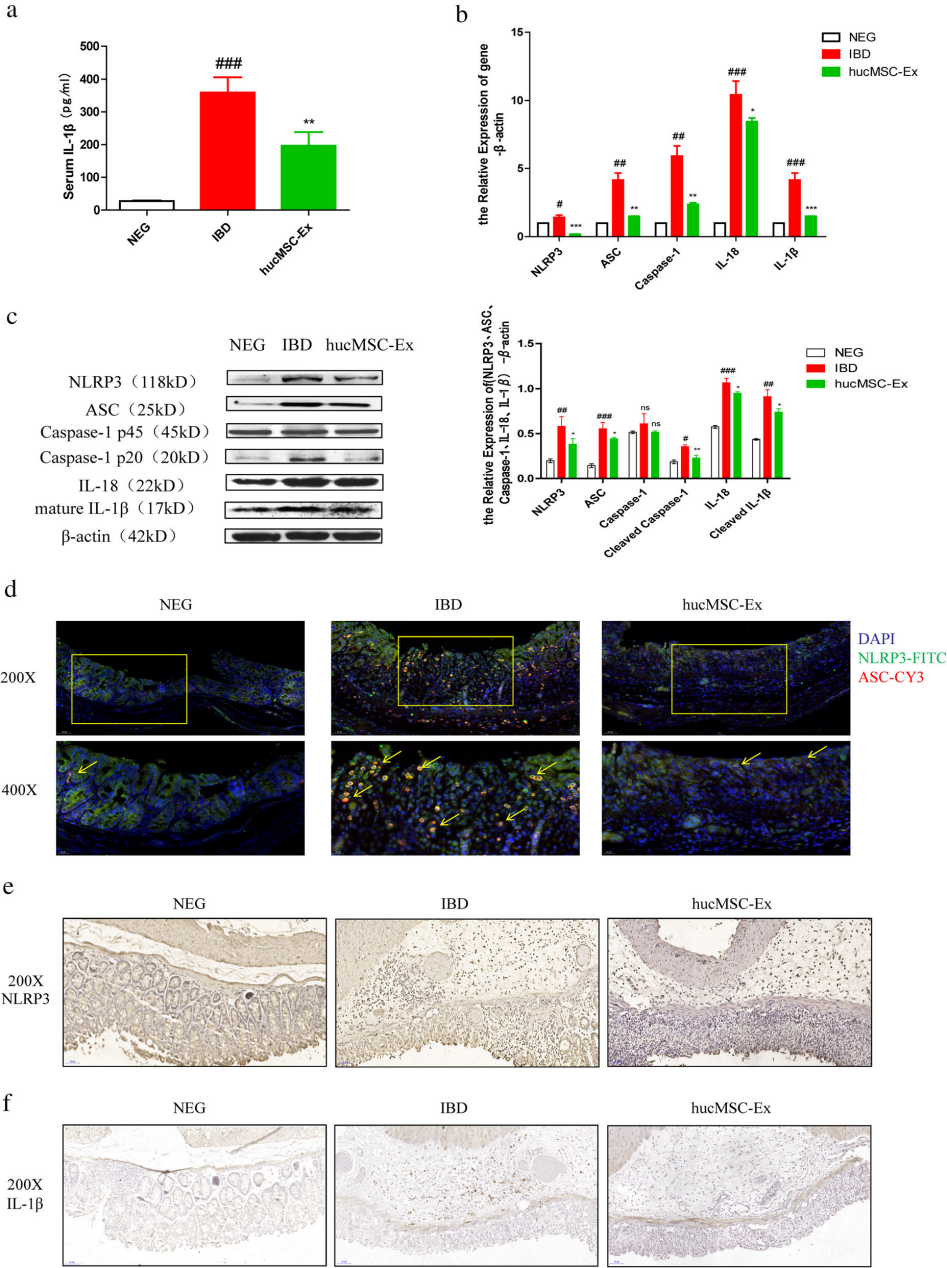
hucMSC-derived exosomes protect against DSS-induced colitis via inhibiting NLRP3 inflammasomes. **a** The quantitative analysis of mouse serum IL-1β (pg ml^−1^) by ELISA. **b** QRT-PCR analysis of the mRNA expression level of NLRP3 inflammasome-related molecules in mouse colon tissues. **c** Western blot analysis of the protein expression level of NLRP3 inflammasome-related molecules in mouse colon tissues and its grayscale scanning analysis. **d** Representative images of IF staining for NLRP3 and ASC on sections of mouse colon tissues (200×, scale bar = 50 μm; 400×, scale bar = 20 μm). **e** IHC analysis of NLRP3 expression in mouse colon tissues (200×, scale bar = 50 μm). **f** IHC analysis of IL-1β expression in the mouse colon tissues (200×, scale bar = 50 μm). #*P* < 0.05, ##*P* < 0.01, ###*P* < 0.001 vs Ctrl by ANOVA; *P < 0.05, **P < 0.01, ***P < 0.001 vs LPS + NIG by ANOVA

We explored the mechanism for hucMSC-derived exosome induced-repair concerning the activation of NLRP3 inflammasomes in mouse colon. The serum cytokine IL-1β was used to evaluate systemic inflammation reactivity. The result of ELISA showed that compared with mice in the IBD group, the level of serum IL-1β in the hucMSC-Ex group significantly decreased (Fig. 3A). RT-PCR and western blot results revealed that the expression of NLRP3 inflammasome-related molecules (NLRP3, ASC, Caspase-1, IL-18, IL-1β) were remarkably decreased in the hucMSC-Ex group compared with the IBD group (Fig. 3B, C). Western blot analysis further showed that hucMSC-derived exosome treatment decreased cleaved Caspase-1 protein. For a more objective and direct observation of the inflammasome activation, IF staining of mouse colon sections was performed, which showed that hucMSC-derived exosomes downregulated the number of cells in which NLRP3 and ASC were both positive in the colon (Fig. 3D). Moreover, NLRP3 synthesis was lower after hucMSC-derived exosome administration as demonstrated by IHC (Fig. 3E), resulting in reduced expression of colonic IL-1β, since it depends on NLRP3 mediation (Fig. 3F). These findings sum up the indication that hucMSC-derived exosomes inhibit the activation of NLRP3 inflammasomes to protect against mouse colitis.

**Fig. 4:**
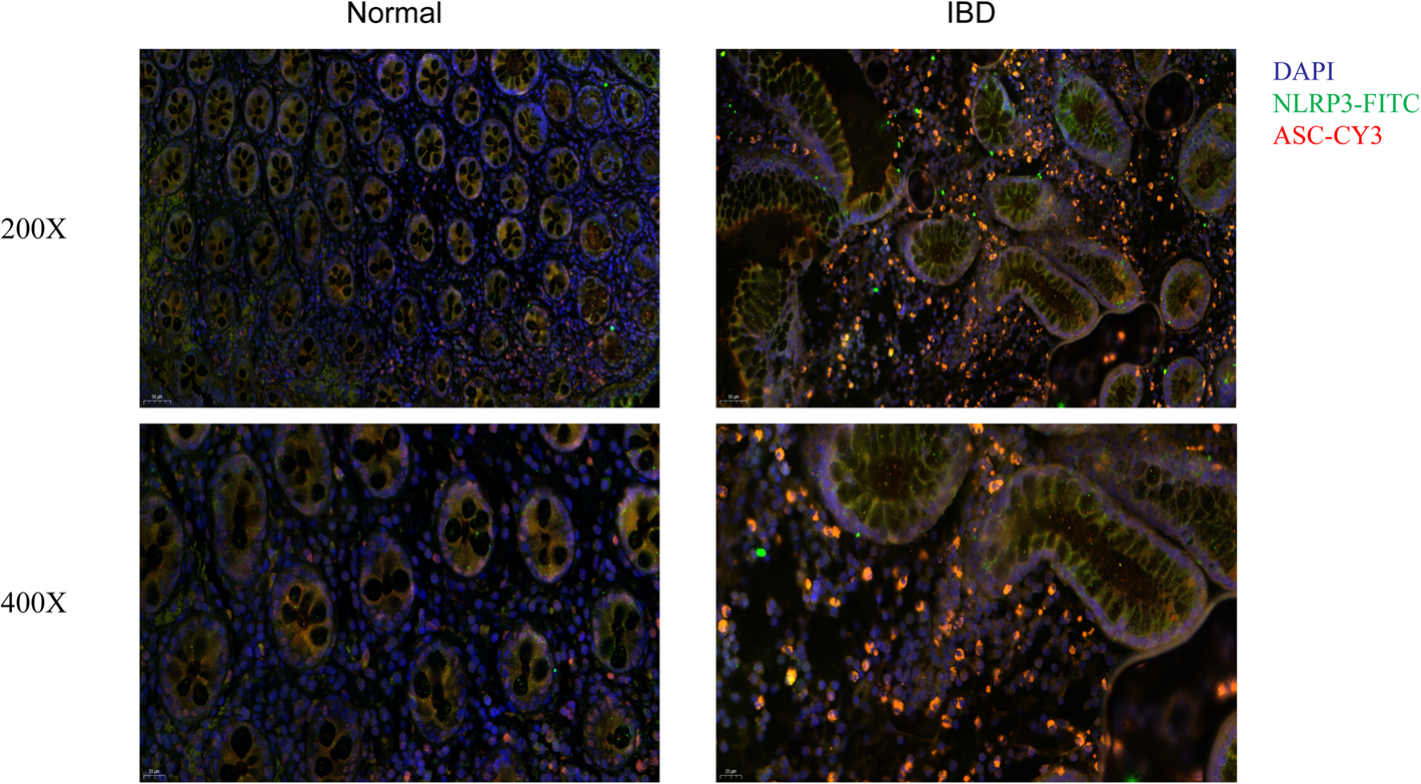
Increased expression of NLRP3 inflammasomes in IBD patients’ colon tissues compared with healthy controls. Representative images of IF staining for NLRP3 and ASC (200×, scale bar = 50 μm; 400×, scale bar = 20 μm)

The analysis of intestinal tissue specimens from healthy volunteers and IBD patients from Nanjing Jiangning People’s Hospital via IF staining revealed increased co-expression of NLRP3 and ASC in colons of IBD patients compared with healthy controls (Fig. 4). This provides clinical implication of potential treatment target of NLRP3 inflammasomes in IBD.

### hucMSC-derived exosomes downregulate the activation of NLRP3 inflammasomes in macrophages

**Fig. 5:**
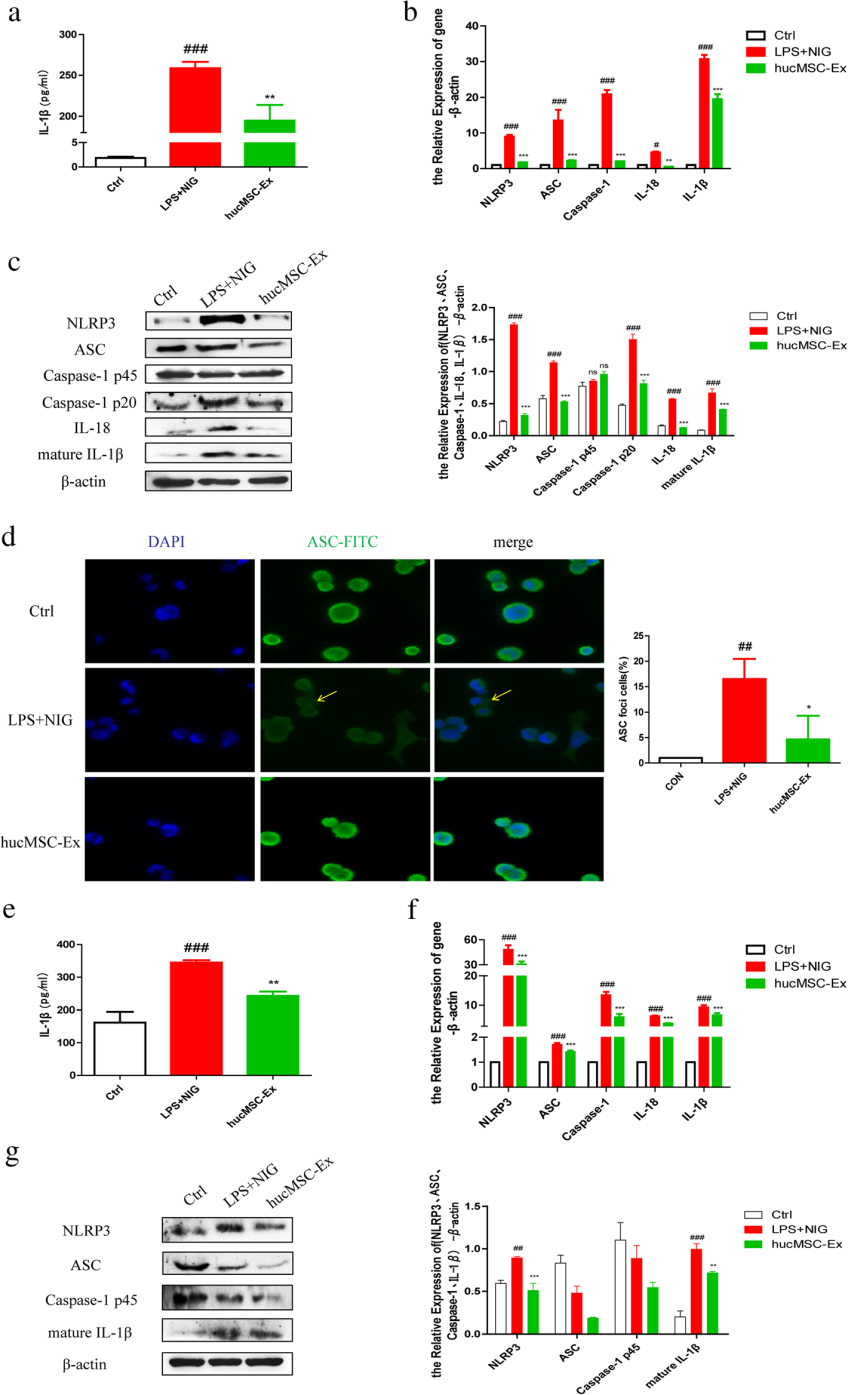
hucMSC-derived exosomes decrease the activation of NLRP3 inflammasomes in macrophages. **a** The quantitative analysis of IL-1β (pg ml^−1^) in THP-1 cell supernatant by ELISA. **b** QRT-PCR analysis of the mRNA expression level of NLRP3 inflammasome-related molecules in THP-1 cells. **c** Western blot analysis of the protein expression level of NLRP3 inflammasome-related molecules in THP-1 cell lysate and grayscale scanning analysis. **d** IF analysis of ASC oligomerization in THP-1 cells (scale bar = 50 μm) and the statistical analysis of the percentage of ASC foci cells. **e** The quantitative analysis of IL-1β (pg ml^−1^) in MPM supernatant by ELISA. **f** QRT-PCR analysis of the mRNA expression level of NLRP3 inflammasome-related molecules in MPMs. **g** Western blot analysis of the protein expression level of NLRP3 inflammasome-related molecules in MPMs lysate and grayscale scanning analysis. #*P* < 0.05, ##*P* < 0.01, ###*P* < 0.001 vs Ctrl by ANOVA; *P < 0.05, **P < 0.01, ***P < 0.001 vs LPS + NIG by ANOVA

Macrophages play a variety of roles to maintain intestinal homeostasis and are involved in the onset and development of IBD [30]. Therefore, we examined the expression of NLRP3 inflammasome-related molecules concerning macrophages. The results showed the treatment of LPS-primed THP-1 cells with 200 μg ml^−1^ hucMSC-derived exosomes before the activation of NLRP3 with Nigericin resulted in a significant reduction in IL-1β release (Fig. 5A) and NLRP3 inflammasome activation (Fig. 5B, C). IF analysis of THP-1 cells showed large spherical intracellular ASC speck formation, following the stimulation of LPS and Nigericin, while hucMSC-derived exosomes reduced the ASC speck formation (Fig. 5D). MPMs were extracted to repeat the cell experiments on the immune regulation of hucMSC-derived exosomes on macrophages. hucMSC-derived exosomes downregulated mouse peritoneal macrophage IL-1β release even after NLRP3 inflammasome activation (Fig. 5E). We also observed similar RT-PCR results as the THP-1 cell experiments, where the relative expressions of NLRP3, ASC, Caspase-1, IL-18, and IL-1β were significantly decreased in the macrophages with hucMSC-derived exosome administration (Fig. 5F). Meanwhile, western blot results showed reduced expression of cell protein NLRP3 and IL-1β after hucMSC-derived exosome treatment (Fig. 5G).

### hucMSC-derived exosomes delay cell pyroptosis

**Fig. 6:**
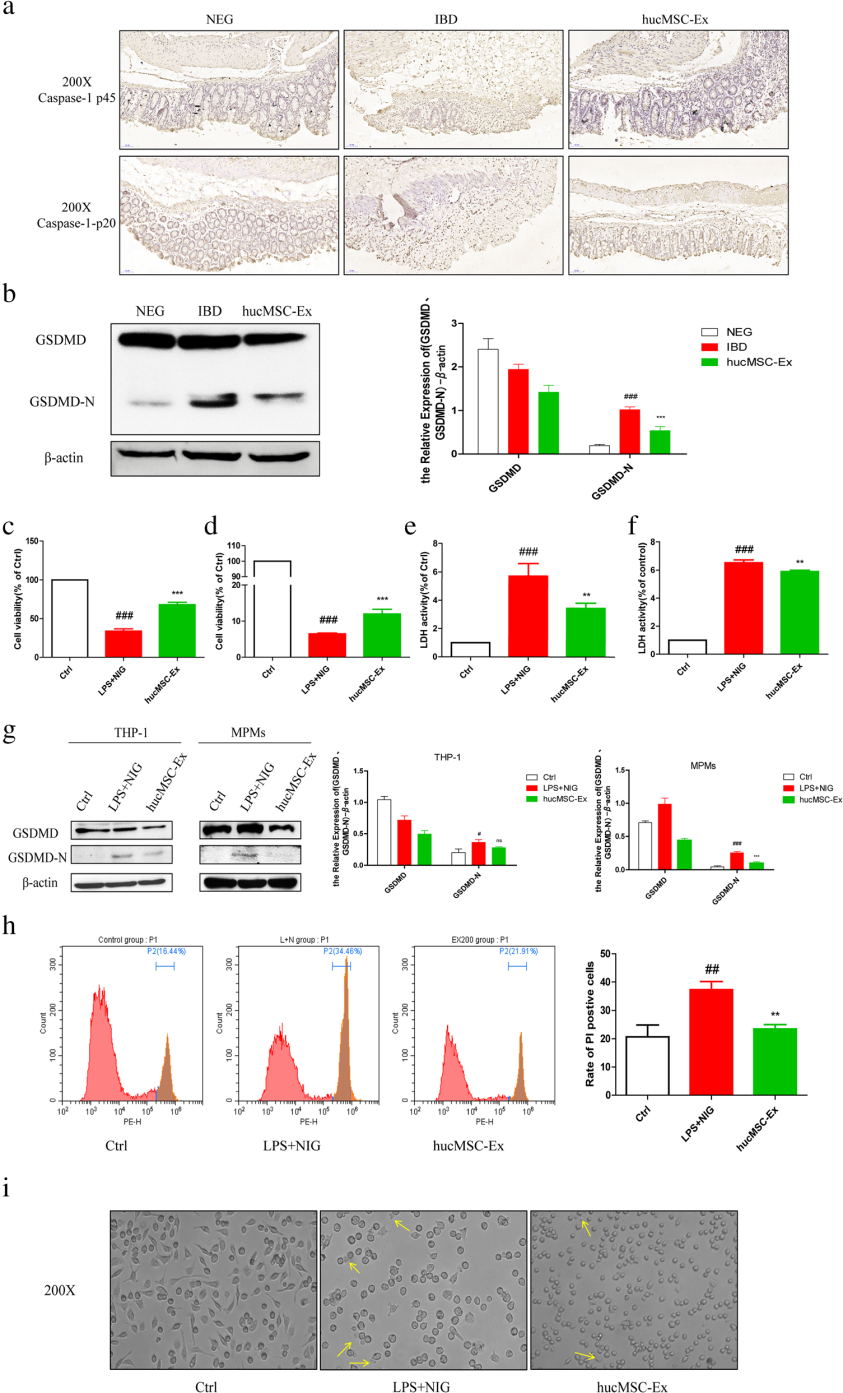
hucMSC-derived exosomes reduce cell pyroptosis by decreasing the activation of NLRP3 inflammasomes both in vivo and in vitro. **a** IHC analysis of Caspase-1 p45 and Caspase-1 p20 expression in mouse colon tissues (200×, scale bar = 50 μm). **b** Western blot analysis of GSDMD protein levels in mouse colon tissues and the grayscale scanning analysis. **c** CCK8 assay analysis of THP-1 cell viability. **d** CCK8 assay analysis of MPM viability. **e** LDH activity analysis of THP-1 cell supernatant. **f** LDH activity analysis of MPM supernatant. **g** Identification of PI-positive THP-1 cells by flow cytometry. **h** Western blot analysis of the protein expression level of GSDMD and cleaved GSDMD fragment in both THP-1 cell lysate and MPM lysate and the grayscale scanning analysis. **i** Imaging assay of pyroptosis in MPMs treated as indicated. #*P* < 0.05, ##*P* < 0.01, ###*P* < 0.001 vs Ctrl by ANOVA; *P < 0.05, **P < 0.01, ***P < 0.001 vs LPS + NIG by ANOVA

The assembly of NLRP3 inflammasome leads to the release of pro-inflammatory cytokines IL-18 and IL-1β which depend on Caspase-1, as well as gasdermin D (GSDMD)-mediated pyroptosis [18]. IHC was performed to observe the expression of Caspase-1 p45 and its mature form Caspase-1 p20 in the mouse colon. The results showed that colorectal tissue in the hucMSC-Ex group presented a weak expression of Caspase-1 compared to the strong expression in the IBD group (Fig. 6A). GSDMD is a substrate of Caspase-1 and the executioner that triggers cell pyroptotic cell death [19]. Western blot analysis of mouse colon mucosal protein showed that hucMSC-exosome therapy decreased the cleavage of GSDMD, contributing to the reduced degree of colon mucosal cell pyroptosis after DSS damage (Fig. 6B). To further confirm that hucMSC-derived exosomes can regulate macrophage pyroptosis, in vitro cell experiments were carried out. hucMSC-derived exosomes increased cell viability after NLRP3 inflammasome activation in cell counting kit-8 experiments of THP-1 cells (Fig. 6C) and MPMs (Fig. 6D), as well as a reduction in LDH release (Fig. 6E, F), which is used as a measure of pyroptosis. Western blot showed that hucMSC-derived exosomes blocked the cleavage of GSDMD to active N-terminal fragment (Fig. 6H). Flow cytometry analysis of THP-1 cells stimulated with LPS and Nigericin indicated fewer PI-positive THP-1 cells in the hucMSC-derived exosome-treated group than those in the untreated group, which was statistically significant (Fig. 6G). Microscopic observation of pyroptosis in MPMs showed swollen cells with unclear membrane edges after NLRP3 inflammasome stimulus, while hucMSC-derived exosomes inhibited these cell morphological changes (Fig. 6I).

### miR-378a-5p in hucMSC-derived exosomes targets NLRP3 to hinder the assembly of inflammasomes

**Fig. 7:**
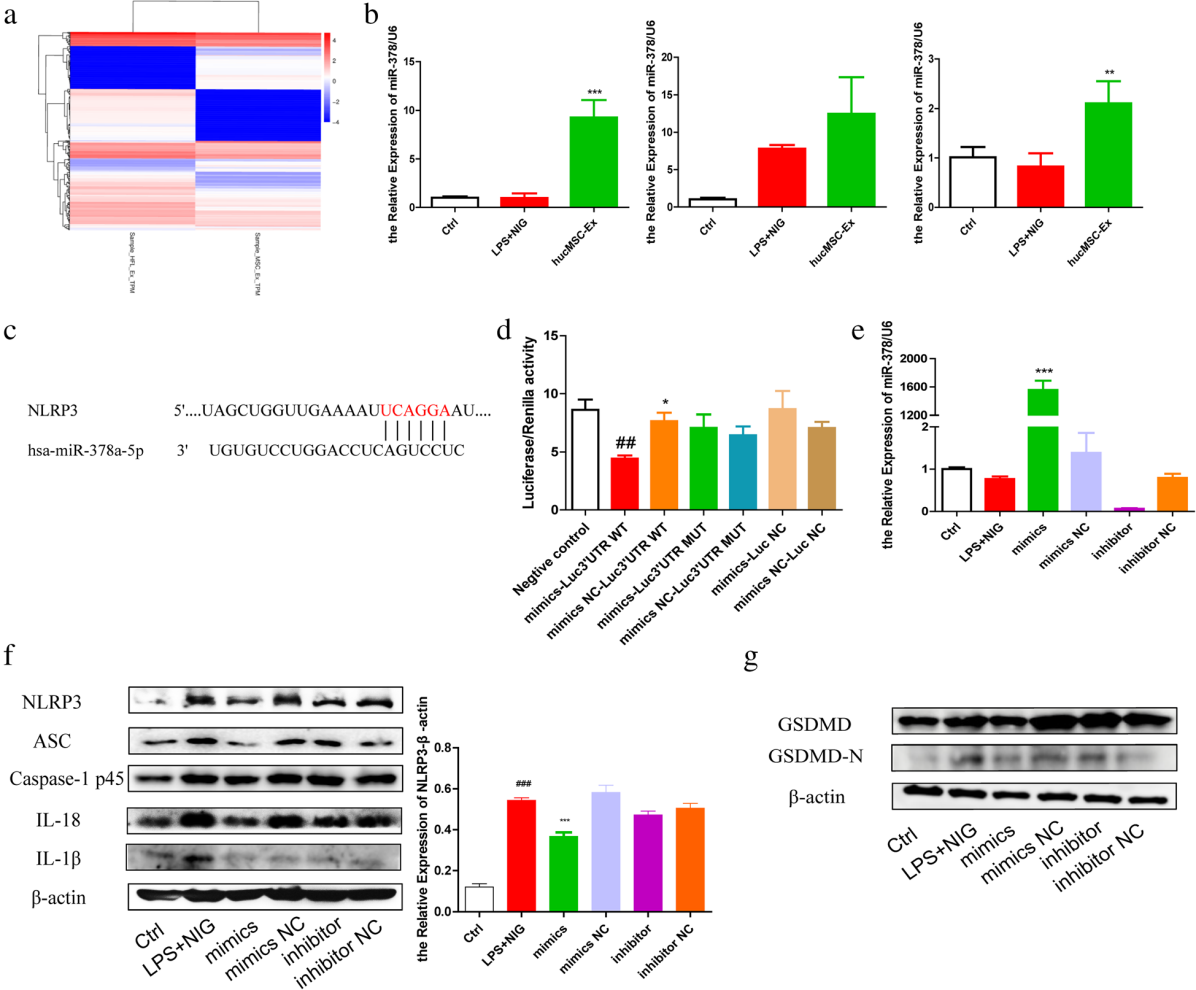
miR-378a-5p is a key molecule in the inhibitory effect of hucMSC-exosomes on NLRP3 inflammasome activation. **a** Sequence results of miRNAs in hucMSC-derived exosomes and HFL1-derived exosomes. **b** QRT-PCR analysis of miR-378a-5p expression levels in THP-1 cells treated as indicated. **c** Binding sites between NLRP3 and miR-378a-5p. **d** Dual-luciferase reporter gene detection of the targeting relationship between NLRP3 and miR-378a-5p. **e** QRT-PCR analysis of the miR-378a-5p in THP-1 cells after transfection. **f** Western blot analysis of the protein expression level of NLRP3 inflammasome-related molecules in THP-1 cell lysate after transfection and the grayscale scanning analysis of NLRP3. **g** Western blot analysis of the protein expression level of GSDMD and cleaved GSDMD fragment in THP-1 cell lysate after transfection. #*P* < 0.05, ##*P* < 0.01, ###*P* < 0.001 vs Ctrl by ANOVA; *P < 0.05, **P < 0.01, ***P < 0.001 vs LPS + NIG (mimics Luc3′UTR WT) by ANOVA

To further explore the mechanism of hucMSC-derived exosomes’ inhibitory effect on NLRP3 inflammasomes, Illumina Hiseq (Oebiotech, OE2015H1459) was used to sequence hucMSC-derived exosomes and HFL1-derived exosomes for comparison (Fig. 7A, source: Key Laboratory of Medical Science and Laboratory Medicine of Jiangsu Province, School of Medicine, Jiangsu University) and prediction of potential miRNA targeting NLRP3 through http://www.targetscan.org. The result showed that miR-378a-5p is stably and highly expressed in hucMSC-derived exosomes and extraction of cells treated with hucMSC-derived exosomes at a different time (Fig. 7B). The probable 3′UTR binding site between miR-378a-5p and NLRP3 mRNA was predicted (Fig. 7C). To confirm that miR-378a-5p binds to NLRP3 mRNA directly, we established luciferase reporters containing the WT and Mut types of 3′UTR in NLRP3 mRNA. As has been expected, the ratio of the two luciferase activities of the WT in the miR-378a-5p mimics group was significantly reduced, while there was no significant difference in the MUT (Fig. 7D). THP-1 cells were transfected with miR-378a-5p mimics, mimics NC, inhibitor, and inhibitor NC for 12 h, and RT-PCR results showed that miR-378a-5p was significantly overexpressed in the miR-378a-5p mimics group and significantly decreased in the miR-378a-5p inhibitors group (Fig. 7E). With a successful transfection, we stimulated THP-1 cells with LPS and Nigericin. Western blot results indicated that miR-378a-5p mimics significantly inhibited the expression of NLRP3 and reduced ASC and IL-18 proteins (Fig. 7F). GSDMD cleavage was also decreased after miR-378a-5p mimic transfection (Fig. 7G). These findings reveal that miR-378a-5p is the key molecule in hucMSC-derived exosomes that interacts with NLRP3 to inhibit NLRP3 inflammasomes assembly and reduces pyroptosis.

## Discussion

IBD significantly impacts the patient’s quality of life [31] and imposes a significant fiscal and resource burden on healthcare systems [32]. Moreover, recurrent and chronic intestinal inflammation has been identified as one of the major risk factors for colorectal cancer [33]; hence, IBD interventions are urgent and necessary. Overactivation of inflammatory and autoimmune responses damages the intestinal mucosal barrier, regarded as one of the vital causes in the development of IBD [34]. Therefore, target suppression of excessive inflammatory activation serves as a promising therapy for IBD.

Intensive investigations have been performed to elucidate the function of NLRP3 inflammasomes in IBD, which is still controversial. The exact role of NLRP3 inflammasomes in IBD seems to have both protective and pathogenic effects [35, 36]. On one hand, NLRP3 inflammasomes prevent colonic damage as reported by Zaki et al. [37], who showed that mice deficient in NLRP3 or ASC and Caspase-1 had increased mortality and were highly susceptible to DSS-induced and severe experimental colitis. Other studies have demonstrated the same protective effects in IBD [38, 39]. On the other hand, hyperactivation of NLRP3 inflammasomes aggravates colonic inflammation. NLRP3^(−/−)^ mice exhibited resistance to colon mucosal damage and relieved colitis in the DSS or TNBS (2,4,6-trinitrobenzene sulfonic acid) model [40–42], and IL-10^(−/−)^ model [43, 44], with reduced mortality. In this process, the cytokines IL-1β and IL-18 appear early in intestinal inflammation and their pro-forms are processed through the Caspase-1-activating multiprotein complex, the NLRP3 inflammasome [42]. Moreover, IL-1β secretion has been shown to be dependent on phagocytosis, lysosomal maturation, cathepsin B and L, and reactive oxygen species (ROS) [41].

In our study, mice with oral administration of DSS had much severer inflammation in colorectal tissues, while the activation of NLRP3 inflammasomes decreased after hucMSC-derived exosome administration. Other sources of exosome such as embryonic stem cells [45] and ginger rhizome [46] have been demonstrated to inhibit TLR4-NLRP3-mediated inflammatory pyroptotic cell death and suppress NLRP3 inflammasome pathway downstream activation, including Caspase-1 autocleavage, IL-1β and IL-18 secretion, and pyroptotic cell death respectively. Intestinal tissue specimens of IBD patients showed NLRP3 inflammasome activation in the colon lesion as previously reported [47]. The increased and aberrant activity of the NLRP3 inflammasomes constitutes a crucial step in the initiation of inflammation and the development of IBD clinical manifestation. Therefore, it is of high importance to medical science to understand the mechanism by which key molecules in hucMSC-derived exosomes retrain the activation of NLRP3 inflammasomes and IL-1β production.

Current therapies for IBD patients are mainly anti-inflammatory and immunosuppressive drugs, which offer poor curative efficacy with severe side effects. Therefore, potent new agents are urgently needed to remedy the deficiency of translational drugs. Exosomes can be used as biomarkers, vaccines, and drug carriers due to their wide biological distribution and excellent biocompatibility, so they can be reasonably modified for the treatment of diseases including IBD as extensively reviewed [48, 49]. Exosomes from multiple kinds of cells can mitigate inflammatory response by downregulating pro-inflammatory cytokines such as IL-1β, TNF-α, inducible nitric oxide synthase (iNOS), cyclooxygenase (COX)-2, monocyte chemoattractant protein (MCP)-1, and upregulating anti-inflammatory cytokines such as IL-10 [50]. MSC-derived exosomes have been reported to protect against myocardial I/R injury, liver fibrosis, retinal injury, diabetes-related complications, limb ischemic, renal injury, pulmonary hypertension, and cutaneous wounding [11, 51]. Our data shows that after hucMSC-derived exosome injection, local and systemic inflammatory symptoms in mice with IBD were relieved. Further analysis revealed that hucMSC-derived exosomes inhibited the expression of NLRP3 inflammasomes and the secretion of pro-inflammatory cytokines in the mouse colon.

Macrophages participate in the activation of various antimicrobial mechanisms and secrete pro-inflammatory factors. For example, the cross-interaction between gut microbiota and macrophages could result in the promotion of intestinal permeability [52] and the activation of NLRP3-dependent pyroptosis in alveolar macrophages contributes to pancreatitis-associated lung injury [53]. Thus, the control of inflammatory macrophage response prevents extensive tissue damage in chronic intestinal inflammation [4]. A study by Liu et al. reported that MSC-derived exosomes colocalized with hepatic macrophages and could reduce the secretion of inflammatory factors by suppressing NLRP3 inflammasome activation in macrophages [54]. The NLRP3 was suppressed via the exosomal miR-17 targeting of thioredoxin interacting protein (TXNIP). Another study found that MSC-derived exosomal miR-410 was a crucial regulator of pyroptosis by directly binding to NLRP3 mRNA to suppress the NLRP3 pathway [55]. Similarly, MSC-derived exosomes effectively reduced NLRP3 inflammasome to ameliorate intervertebral disc degeneration [56] and protected against hypoxia/reoxygenation-induced pyroptosis of cardiomyocytes through the miRNA-100-5P/FOXO3/NLRP3 pathway [57]. Our study is consistent with these findings in that hucMSC-derived exosomes decrease the activation of NLRP3 inflammasomes in macrophages and delay the progress of cell pyroptosis in vitro.

**Fig. 8:**
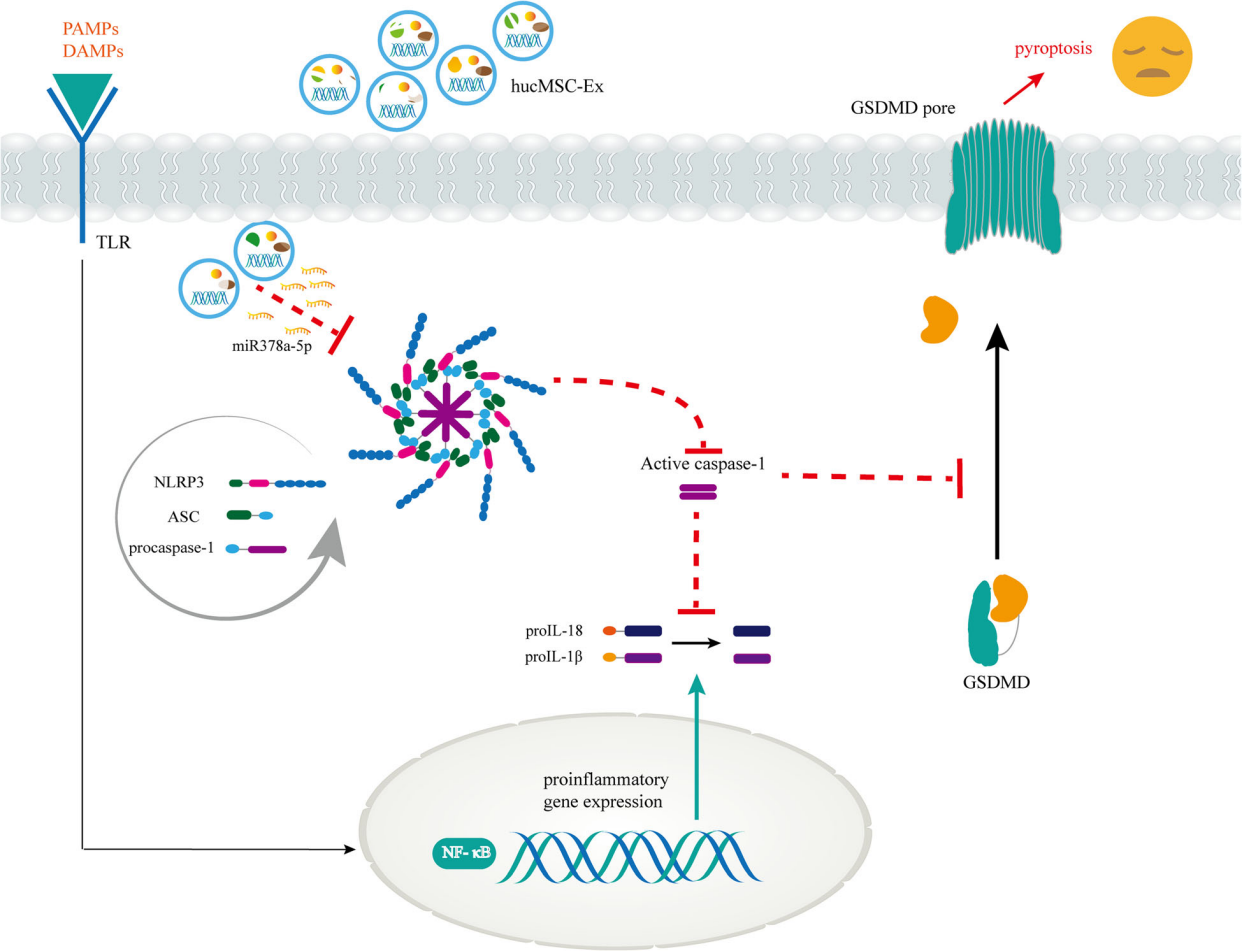
The mechanistic model of miR-378a-5p in hucMSC-derived exosome-mediated NLRP3 inflammasome activation and cell pyroptosis

miRNAs are small non-coding RNAs that inhibit mRNA translation and promote mRNA degradation to modulate target gene expression at the post-transcriptional level [58]. miRNAs play a pivotal role in the regulation of NLRP3 inflammasomes. While some miRNAs activate the NLRP3 inflammasomes, others negatively regulate the activation of the NLRP3 inflammasomes. For example, miR-7 [59], miR-20b [60], miRNA-223 [61, 62], and miRNA-495 [63] relieve tissue injury and inflammation by targeting NLRP3. miRNAs carried in MSC exosomes mediate various cellular activities including angiogenesis and anti-angiogenesis, immunomodulation, anti-fibrosis, and anti-apoptosis [64]. In this current study, the exploration of the mechanism by which hucMSC-derived exosomes hinder the activation of NLRP3 inflammasomes revealed that miR-378a-5p functions as a key molecule (Fig. 8). The hucMSC-derived exosomal miR-378a-5p targeted NLRP3, leading to the blockade of NLRP3 inflammasome assembly and the consequent cleavage of Caspase-1. Reduction of the active Caspase-1 led to decreased maturation of IL-1β and IL-18, with a consequent reduction in the formation of GSDMD pore.

This study is a preliminary exploration of the mechanism of miRNAs in exosomes as targets of NLRP3 inflammasomes and possible therapy for IBD. Deficiencies that exist in the current study include the non-specificity of the mechanism of miR-378a-5p regulating NLRP3, hence the need for further studies to obtain clarity. Moreover, animal rescue experiments need to be carried out to confirm the therapeutic target of the miR-378a-5p/NLRP3 axis.

## Conclusion

The present study shows that hucMSC-derived exosomes effectively inhibit NLRP3 inflammasomes in macrophages and delay cell pyroptosis, contributing to the amelioration of IBD. hucMSC-derived exosomes play a beneficial role in regulating macrophage. NLRP3 inflammasomes provide a novel insight into the use of hucMSC-derived exosome anti-inflammatory activity as a feasible therapy in the clinic. It also provides new ideas and experimental basis for the clinical application of small molecule drugs targeting NLRP3 inflammasome in the treatment of inflammatory diseases.

## Data Availability

All data and materials generated and analyzed during the present study are available from the corresponding author on reasonable request.

## Abbreviations

DAI: Disease activity index
DSS: Dextran sulfate sodium
hucMSCs: Human umbilical cord mesenchymal stem cells
IBD: Inflammatory bowel disease
MPMs: Mouse peritoneal macrophages
NLRP3: The nucleotide-binding domain and leucine-rich repeat-containing family, pyrin domain-containing 3

## References

[1] Marion-Letellier R, Savoye G, Ghosh S (2016). IBD: In food we trust. J Crohns Colitis..

[2] de Souza HS, Fiocchi C (2016). Immunopathogenesis of IBD: current state of the art. Nat Rev Gastroenterol Hepatol..

[3] Kaplan GG (2015). The global burden of IBD: from 2015 to 2025. Nat Rev Gastroenterol Hepatol..

[4] Lissner D, Schumann M, Batra A, Kredel LI, Kuhl AA, Erben U (2015). Monocyte and M1 macrophage-induced barrier defect contributes to chronic intestinal inflammation in IBD. Inflamm Bowel Dis..

[5] Wallace KL, Zheng LB, Kanazawa Y, Shih DQ (2014). Immunopathology of inflammatory bowel disease. World J Gastroenterol..

[6] Medzhitov R (2007). Recognition of microorganisms and activation of the immune response. Nature..

[7] Na YR, Stakenborg M, Seok SH, Matteoli G (2019). Macrophages in intestinal inflammation and resolution: a potential therapeutic target in IBD. Nat Rev Gastroenterol Hepatol..

[8] Hass R, Kasper C, Bohm S, Jacobs R (2011). Different populations and sources of human mesenchymal stem cells (MSC): a comparison of adult and neonatal tissue-derived MSC. Cell Commun Signal..

[9] Asami T, Ishii M, Fujii H, Namkoong H, Tasaka S, Matsushita K (2013). Modulation of murine macrophage TLR7/8-mediated cytokine expression by mesenchymal stem cell-conditioned medium. Mediators Inflamm..

[10] Sahoo S, Losordo DW (2014). Exosomes and cardiac repair after myocardial infarction. Circ Res..

[11] Toh WS, Lai RC, Zhang B, Lim SK (2018). MSC exosome works through a protein-based mechanism of action. Biochem Soc Trans..

[12] Lai P, Weng J, Guo L, Chen X, Du X (2019). Novel insights into MSC-EVs therapy for immune diseases. Biomark Res..

[13] Ma ZJ, Wang YH, Li ZG, Wang Y, Li BY, Kang HY, Wu XY (2019). Immunosuppressive effect of exosomes from mesenchymal stromal cells in defined medium on experimental colitis. Int J Stem Cells..

[14] Liu H, Liang Z, Wang F, Zhou C, Zheng X, Hu T, et al. Exosomes from mesenchymal stromal cells reduce murine colonic inflammation via a macrophage-dependent mechanism. JCI Insight. 2019;4(24). 10.1172/jci.insight.131273 PMC6975270 31689240

[15] Mao F, Wu Y, Tang X, Kang J, Zhang B, Yan Y (2017). Exosomes derived from human umbilical cord mesenchymal stem cells relieve inflammatory bowel disease in mice. Biomed Res Int..

[16] Serafini MA, Mello HF, Pfaffenseller B, Araujo AB, Visioli F, F DACG (2018). Bioactive factors secreted from mesenchymal stromal cells protect the intestines from experimental colitis in a three-dimensional culture. Cytotherapy..

[17] Henderson J, Bhattacharyya S, Varga J, O'Reilly S (2018). Targeting TLRs and the inflammasome in systemic sclerosis. Pharmacol Ther..

[18] Awad F, Assrawi E, Louvrier C, Jumeau C, Georgin-Lavialle S, Grateau G, Amselem S, Giurgea I, Karabina SA (2018). Inflammasome biology, molecular pathology and therapeutic implications. Pharmacol Ther..

[19] Rathinam VAK, Chan FK (2018). Inflammasome, inflammation, and tissue homeostasis. Trends Mol Med..

[20] Yu HB, Finlay BB (2008). The caspase-1 inflammasome: a pilot of innate immune responses. Cell Host Microbe..

[21] Petrilli V, Dostert C, Muruve DA, Tschopp J (2007). The inflammasome: a danger sensing complex triggering innate immunity. Curr Opin Immunol..

[22] He WT, Wan H, Hu L, Chen P, Wang X, Huang Z, Yang ZH, Zhong CQ, Han J (2015). Gasdermin D is an executor of pyroptosis and required for interleukin-1beta secretion. Cell Res..

[23] Afonina IS, Zhong Z, Karin M, Beyaert R (2017). Limiting inflammation-the negative regulation of NF-kappaB and the NLRP3 inflammasome. Nat Immunol..

[24] Chung IC, OuYang CN, Yuan SN, Lin HC, Huang KY, Wu PS, et al. Pretreatment with a heat-killed probiotic modulates the NLRP3 inflammasome and attenuates colitis-associated colorectal cancer in mice. Nutrients. 2019;11(3). 10.3390/nu11030516 PMC6471765 30823406

[25] Qiao C, Xu W, Zhu W, Hu J, Qian H, Yin Q, Jiang R, Yan Y, Mao F, Yang H, Wang X, Chen Y (2008). Human mesenchymal stem cells isolated from the umbilical cord. Cell Biol Int..

[26] Wang G, Yuan J, Cai X, Xu Z, Wang J, Ocansey DKW, Yan Y, Qian H, Zhang X, Xu W, Mao F (2020). HucMSC-exosomes carrying miR-326 inhibit neddylation to relieve inflammatory bowel disease in mice. Clin Transl Med..

[27] Kang J, Zhang Z, Wang J, Wang G, Yan Y, Qian H (2019). hucMSCs attenuate IBD through releasing miR148b-5p to inhibit the expression of 15-lox-1 in macrophages. Mediators Inflamm.

[28] Sun Y, Shi H, Yin S, Ji C, Zhang X, Zhang B, Wu P, Shi Y, Mao F, Yan Y, Xu W, Qian H (2018). Human mesenchymal stem cell derived exosomes alleviate type 2 diabetes mellitus by reversing peripheral insulin resistance and relieving beta-cell destruction. ACS Nano..

[29] Pineda-Torra I, Gage M, de Juan A, Pello OM (2015). Isolation, culture, and polarization of murine bone marrow-derived and peritoneal macrophages. Methods Mol Biol..

[30] Bain CC, Mowat AM (2014). Macrophages in intestinal homeostasis and inflammation. Immunol Rev..

[31] Latella G, Rogler G, Bamias G, Breynaert C, Florholmen J, Pellino G, Reif S, Speca S, Lawrance IC (2014). Results of the 4th scientific workshop of the ECCO (I): pathophysiology of intestinal fibrosis in IBD. J Crohns Colitis..

[32] Windsor JW, Kaplan GG (2019). Evolving epidemiology of IBD. Curr Gastroenterol Rep..

[33] Parian A, Koh J, Limketkai BN, Eluri S, Rubin DT, Brant SR, Ha CY, Bayless TM, Giardiello F, Hart J, Montgomery E, Lazarev MG (2016). Association between serrated epithelial changes and colorectal dysplasia in inflammatory bowel disease. Gastrointest Endosc..

[34] Nanini HF, Bernardazzi C, Castro F, de Souza HSP (2018). Damage-associated molecular patterns in inflammatory bowel disease: from biomarkers to therapeutic targets. World J Gastroenterol..

[35] Zhen Y, Zhang H (2019). NLRP3 inflammasome and inflammatory bowel disease. Front Immunol..

[36] Tourkochristou E, Aggeletopoulou I, Konstantakis C, Triantos C (2019). Role of NLRP3 inflammasome in inflammatory bowel diseases. World J Gastroenterol..

[37] Zaki MH, Boyd KL, Vogel P, Kastan MB, Lamkanfi M, Kanneganti TD (2010). The NLRP3 inflammasome protects against loss of epithelial integrity and mortality during experimental colitis. Immunity..

[38] Itani S, Watanabe T, Nadatani Y, Sugimura N, Shimada S, Takeda S, Otani K, Hosomi S, Nagami Y, Tanaka F, Kamata N, Yamagami H, Tanigawa T, Shiba M, Tominaga K, Fujiwara Y, Arakawa T (2016). NLRP3 inflammasome has a protective effect against oxazolone-induced colitis: a possible role in ulcerative colitis. Sci Rep..

[39] Dupaul-Chicoine J, Yeretssian G, Doiron K, Bergstrom KS, McIntire CR, LeBlanc PM (2010). Control of intestinal homeostasis, colitis, and colitis-associated colorectal cancer by the inflammatory caspases. Immunity..

[40] Higashimori A, Watanabe T, Nadatani Y, Takeda S, Otani K, Tanigawa T, Yamagami H, Shiba M, Tominaga K, Fujiwara Y, Arakawa T (2016). Mechanisms of NLRP3 inflammasome activation and its role in NSAID-induced enteropathy. Mucosal Immunol..

[41] Bauer C, Duewell P, Mayer C, Lehr HA, Fitzgerald KA, Dauer M, Tschopp J, Endres S, Latz E, Schnurr M (2010). Colitis induced in mice with dextran sulfate sodium (DSS) is mediated by the NLRP3 inflammasome. Gut..

[42] Bauer C, Duewell P, Lehr HA, Endres S, Schnurr M (2012). Protective and aggravating effects of Nlrp3 inflammasome activation in IBD models: influence of genetic and environmental factors. Digestive diseases (Basel, Switzerland).

[43] Zhang J, Fu S, Sun S, Li Z, Guo B (2014). Inflammasome activation has an important role in the development of spontaneous colitis. Mucosal Immunol..

[44] Liu L, Dong Y, Ye M, Jin S, Yang J, Joosse ME, Sun Y, Zhang J, Lazarev M, Brant SR, Safar B, Marohn M, Mezey E, Li X (2017). The pathogenic role of NLRP3 inflammasome activation in inflammatory bowel diseases of both mice and humans. J Crohns Colitis..

[45] Tavakoli Dargani Z, Singla DK (2019). Embryonic stem cell-derived exosomes inhibit doxorubicin-induced TLR4-NLRP3-mediated cell death-pyroptosis. American journal of physiology Heart and circulatory physiology..

[46] Chen X, Zhou Y, Yu J (2019). Exosome-like nanoparticles from ginger rhizomes inhibited NLRP3 inflammasome activation. Molecular pharmaceutics..

[47] Lazaridis LD, Pistiki A, Giamarellos-Bourboulis EJ, Georgitsi M, Damoraki G, Polymeros D, Dimitriadis GD, Triantafyllou K (2017). Activation of NLRP3 inflammasome in inflammatory bowel disease: differences between Crohn’s disease and ulcerative colitis. Dig Dis Sci..

[48] Ocansey DKW, Zhang L, Wang Y, Yan Y, Qian H, Zhang X, Xu W, Mao F (2020). Exosome-mediated effects and applications in inflammatory bowel disease. Biol Rev Camb Philos Soc..

[49] Wu P, Zhang B, Ocansey DKW, Xu W, Qian H (2021). Extracellular vesicles: a bright star of nanomedicine. Biomaterials..

[50] Wu P, Zhang B, Shi H, Qian H, Xu W (2018). MSC-exosome: a novel cell-free therapy for cutaneous regeneration. Cytotherapy..

[51] Toh WS, Lai RC, Hui JHP, Lim SK (2017). MSC exosome as a cell-free MSC therapy for cartilage regeneration: implications for osteoarthritis treatment. Semin Cell Dev Biol..

[52] Isaacs-Ten A, Echeandia M, Moreno-Gonzalez M, Brion A, Goldson A, Philo M (2020). Intestinal microbiome-macrophage crosstalk contributes to cholestatic liver disease by promoting intestinal permeability in mice. Hepatology..

[53] Wu XB, Sun HY, Luo ZL, Cheng L, Duan XM, Ren JD (1866). Plasma-derived exosomes contribute to pancreatitis-associated lung injury by triggering NLRP3-dependent pyroptosis in alveolar macrophages. Biochim Biophys Acta Mol Basis Dis..

[54] Liu Y, Lou G, Li A, Zhang T, Qi J, Ye D, Zheng M, Chen Z (2018). AMSC-derived exosomes alleviate lipopolysaccharide/d-galactosamine-induced acute liver failure by miR-17-mediated reduction of TXNIP/NLRP3 inflammasome activation in macrophages. EBioMedicine..

[55] Zhang J, Zhang J, Zhang Y, Liu W, Ni W, Huang X, Yuan J, Zhao B, Xiao H, Xue F (2020). Mesenchymal stem cells-derived exosomes ameliorate intervertebral disc degeneration through inhibiting pyroptosis. Journal of cellular and molecular medicine..

[56] Xia C, Zeng Z, Fang B, Tao M, Gu C, Zheng L, Wang Y, Shi Y, Fang C, Mei S, Chen Q, Zhao J, Lin X, Fan S, Jin Y, Chen P (2019). Mesenchymal stem cell-derived exosomes ameliorate intervertebral disc degeneration via anti-oxidant and anti-inflammatory effects. Free Radic Biol Med..

[57] Liang C, Liu Y, Xu H, Huang J, Shen Y, Chen F (2020). Exosomes of human umbilical cord MSCs protect against hypoxia/reoxygenation-induced pyroptosis of cardiomyocytes via the miRNA-100-5p/FOXO3/NLRP3 pathway. Front Bioeng Biotechnol..

[58] Correia de Sousa M, Gjorgjieva M, Dolicka D, Sobolewski C, Foti M. Deciphering miRNAs’ action through miRNA editing. Int J Mol Sci. 2019;20(24). 10.3390/ijms20246249 PMC6941098 31835747

[59] Zamani P, Oskuee RK, Atkin SL, Navashenaq JG, Sahebkar A (2020). MicroRNAs as important regulators of the NLRP3 inflammasome. Prog Biophys Mol Biol..

[60] Zhao J, Wang H, Dong L, Sun S, Li L (2019). miRNA-20b inhibits cerebral ischemia-induced inflammation through targeting NLRP3. Int J Mol Med..

[61] Neudecker V, Haneklaus M, Jensen O, Khailova L, Masterson JC, Tye H, Biette K, Jedlicka P, Brodsky KS, Gerich ME, Mack M, Robertson AAB, Cooper MA, Furuta GT, Dinarello CA, O’Neill LA, Eltzschig HK, Masters SL, McNamee EN (2017). Myeloid-derived miR-223 regulates intestinal inflammation via repression of the NLRP3 inflammasome. J Exp Med..

[62] Sha R, Zhang B, Han X, Peng J, Zheng C, Zhang F, Huang X (2019). Electroacupuncture alleviates ischemic brain injury by inhibiting the miR-223/NLRP3 pathway. Med Sci Monit..

[63] Ying Y, Mao Y, Yao M (2019). NLRP3 inflammasome activation by microRNA-495 promoter methylation may contribute to the progression of acute lung injury. Mol Ther Nucleic Acids..

[64] Katsuda T, Ochiya T (2015). Molecular signatures of mesenchymal stem cell-derived extracellular vesicle-mediated tissue repair. Stem Cell Res Ther..

